# Radiofrequency Ablation of Typical Atrial Flutter via Right Jugular Vein due to Bilateral Obstructed Iliac Veins in a Patient with Dilated Cardiomyopathy

**DOI:** 10.1155/2015/401580

**Published:** 2015-01-27

**Authors:** Tolga Aksu, Tumer Erdem Guler, Sukriye Ebru Golcuk, Kazım Serhan Ozcan, Ismail Erden

**Affiliations:** ^1^Department of Cardiology, Kocaeli Derince Education and Research Hospital, 41500 Kocaeli, Turkey; ^2^Department of Cardiology, Istanbul Medical Faculty, Istanbul University, 34000 Istanbul, Turkey

## Abstract

Ablation of cavotricuspid isthmus (CTI) is the gold standard method in the treatment of isthmus dependent atrial flutter (AFl). Venous access was obtained usually via right or left femoral veins. In rare cases of obstruction of iliofemoral veins, ablation of CTI can be performed only through the superior approach. We present a 74-year-old woman of typical AFl and dilated cardiomyopathy that was ablated through the right jugular vein because of obstruction of the left and the right iliac veins. This is the first report of successful ablation of CTI in a patient with dilated cardiomyopathy via superior approach.

## 1. Introduction

Typical atrial flutter (AFl) is characterized by a macroreentry circuit around the tricuspid annulus; this circuit contains a propagating wave and an excitable gap. The cavotricuspid isthmus (CTI) is a critical component of the circuit; its radiofrequency catheter ablation (RFCA) is the gold standard technique available to block that circuit [1–3].

The central part of the CTI is a main site for creating the linear lesion, because it has a shorter length and thinner myocardium. In standard approach, the procedure is performed via femoral vein through the inferior vena cava (IVC). In rare cases of obstruction of IVC or iliofemoral veins, ablation of CTI can be performed only by superior approach via right internal jugular or subclavian veins. Ablation of CTI via superior approach may be challenging because it does not follow the natural curve to “drag” toward the IVC for creating the linear lesion. We present a case of AFl that was ablated through the right jugular veins due to obstruction of bilaterally iliofemoral veins.

## 2. Case Report

A 74-year-old woman was referred to our cardiology department for ablation of symptomatic, drug-resistant AFl. She has been already under treatment with high dose calcium channel blocker (diltiazem 120 mg bid) and digoxin. Tachycardia ECG revealed sawtooth pattern in leads II, III, and AVF, providing the reasonable diagnosis of typical AFL. Transthoracic echocardiography revealed a severely impaired left ventricular contractility (ejection fraction: 30% calculated according to Simpson's rule); there were no relevant valvular pathologies.

Due to the highly symptomatic medically uncontrolled fast ventricular response of AFL, ablation of CTI was decided. All antiarrhythmic drugs were discontinued more than 7 days before the ablation procedure.

**Figure 1:**
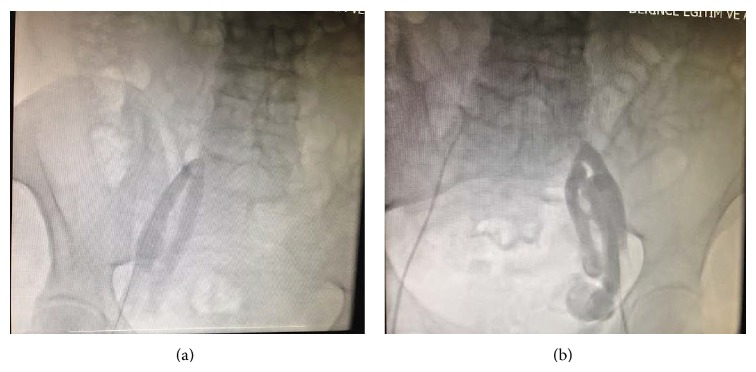
(a) Total occlusion of the right femoral vein seen after contrast injection from right femoral vein. (b) Total occlusion of the left iliac vein seen after contrast injection from left femoral vein.

**Figure 2:**
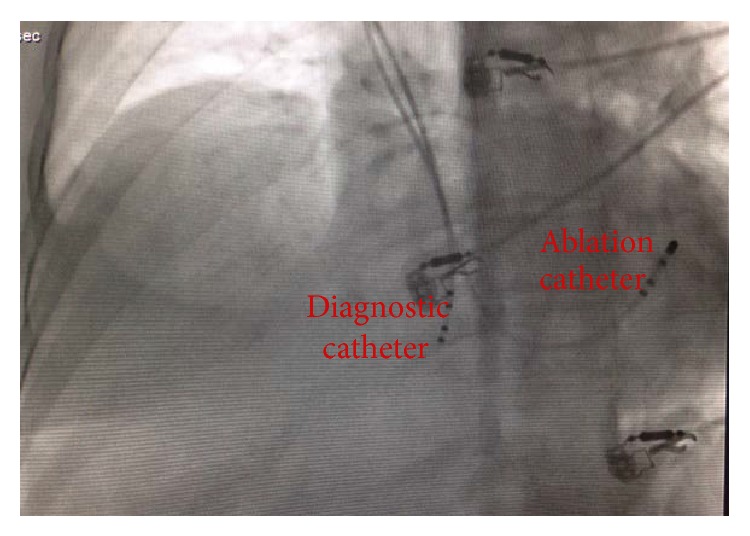
Modified left anterior oblique view. The ablation catheter and diagnostic catheter are in coronary sinus and on ventricular aspect of cavotricuspid isthmus, respectively.

A right jugular/left subclavian vein access was chosen, as the presence of a bilateral obstruction in the level of iliac veins made inferior approach impossible (Figures 1(a) and 1(b)). After placing two 6F sheaths and one 7F sheath in the right jugular vein, 4-pole fix-curved diagnostic catheters were advanced through the 6F sheaths into the right atrium. Subsequently, a 4-pole 4 mm tip ablation catheter was advanced over the 7F subclavian sheath to the ventricular margin of the CTI zone (Figure 2). All measurements were performed with a Cardiolab system (Prucka Engineering). To reduce the fluoroscopy time, we decided to perform the ablation by using electroanatomical mapping. So we used 3D mapping by Ensite NavX system (St. Jude Medical, Inc., St. Paul, MN, USA).

**Figure 3:**
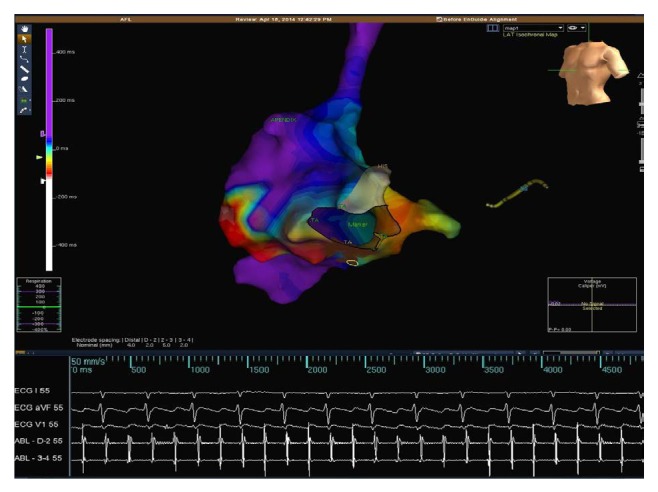
3D mapping of the left atrium using NavX system (St. Jude Medical, St. Paul, MN, USA) was shown during atrial flutter. Isochronal activation map constructed during tachycardia using coronary sinus atrial signal as a reference. The color coded display of the activation time in the right atrium measured relative to the reference point with red being early and blue being late. Activation map shows that activation propagates with counterclockwise route. At the bottom of the figure, intracardiac electrogram recording of the clinical tachycardia is seen.

The activation mapping of right atrium was recorded using the ablation catheter. The intracardiac signals and activation mapping revealed counterclockwise typical AFl (Figure 3) and positive concealed entrainment verified that the tachycardia was isthmus dependent. Ablation of CTI was performed using a point-by-point approach for 60 s at each point with a power limit of 50 W and a target temperature of 55°C.

**Figure 4:**
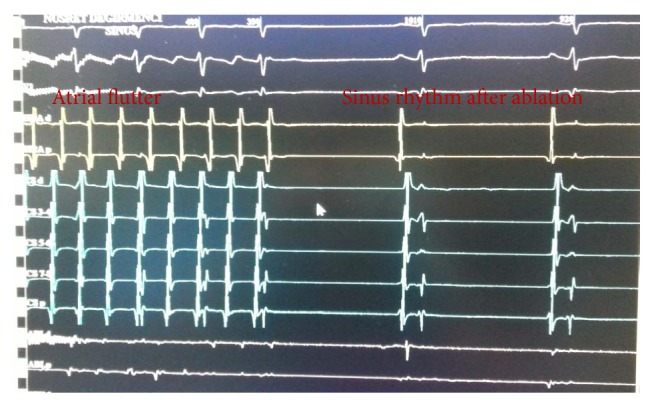
Intracardiac electrograms. Termination of atrial flutter is shown in the last two beats after isthmus ablation. ABL: ablasyon; CS: coronary sinus; HRA: high right atrium.

The radiofrequency applications were started at the ventricular aspect of the tricuspid annulus when a stable electrogram with a small atrial and large ventricular potential was observed. The catheter was withdrawn after each application to find a sharp and large atrial potential, under electroanatomical mapping guidance, to produce a continuous lesion until the IVC was reached. The patient turned to normal sinus rhythm during ablation (Figure 4). With the appropriate maneuvers, the presence of bidirectional block was confirmed [4]. The procedure time was 45 minutes with a total radiofrequency delivery time of 12 minutes and fluoroscopy time of only 5 minutes.

## 3. Discussion

Common AFl is a macroreentrant circuit around the tricuspid annulus, and the transverse conduction block at the CTI is important for the maintenance of sinus rhythm [1]. Ablation of CTI via femoral approach, with complete bidirectional conduction block, is a highly effective treatment for this arrhythmia [2, 3].

In relevant literature, there are only a few reports about ablation of CTI by superior approach. The reason for choosing the superior approach in these cases was the absence of an inferior access or the expected technical difficulties using anomalous inferior paths [5–7]. Using the “superior” approach the catheter has to be pushed instead of being pulled against the CTI. This leads to extreme catheter instability. To decrease fluoroscopy time and to have full tissue contact during superior approach, we decided to perform the ablation by using 3D electroanatomical mapping by “point-by-point” technique. So we did not apply the special catheter stabilization manoeuvres mentioned in previous publications [5–8]. To the best of our knowledge, this is the first report on RFCA of atrial flutter by the superior approach in a patient with dilated cardiomyopathy.

In conclusion, CTI ablation by electroanatomical mapping from jugular vein in patients with no access from IVC seems safe and effective treatment option with an acceptable duration of intervention.
